# Omics analyses in citrus reveal a possible role of RNA translation pathways and Unfolded Protein Response regulators in the tolerance to combined drought, high irradiance, and heat stress

**DOI:** 10.1093/hr/uhad107

**Published:** 2023-05-19

**Authors:** Damián Balfagón, Sara I Zandalinas, Tadeu dos Reis de Oliveira, Claudete Santa-Catarina, Aurelio Gómez-Cadenas

**Affiliations:** Departamento de Biología, Bioquímica y Ciencias Naturales, Av. Sos Baynat s/n. Universitat Jaume I, 46520 Castelló de la Plana, Spain; Departamento de Biología, Bioquímica y Ciencias Naturales, Av. Sos Baynat s/n. Universitat Jaume I, 46520 Castelló de la Plana, Spain; Laboratório de Biologia Celular e Tecidual (LBCT), Centro de Biociências E Biotecnologia (CBB), Universidade Estadual Do Norte Fluminense Darcy Ribeiro (UENF), Av. Alberto Lamego 2000, Campos Dos Goytacazes, RJ, 28013-602, Brazil; Laboratório de Biologia Celular e Tecidual (LBCT), Centro de Biociências E Biotecnologia (CBB), Universidade Estadual Do Norte Fluminense Darcy Ribeiro (UENF), Av. Alberto Lamego 2000, Campos Dos Goytacazes, RJ, 28013-602, Brazil; Departamento de Biología, Bioquímica y Ciencias Naturales, Av. Sos Baynat s/n. Universitat Jaume I, 46520 Castelló de la Plana, Spain

## Abstract

Environmental changes derived from global warming and human activities increase the intensity and frequency of stressful conditions for plants. Multiple abiotic factors acting simultaneously enhance stress pressure and drastically reduce plant growth, yield, and survival. Stress combination causes a specific stress situation that induces a particular plant response different to the sum of responses to the individual stresses. Here, by comparing transcriptomic and proteomic profiles to different abiotic stress combinations in two citrus genotypes, Carrizo citrange (*Citrus sinensis × Poncirus trifoliata*) and Cleopatra mandarin (*Citrus reshni*), with contrasting tolerance to different abiotic stresses, we revealed key responses to the triple combination of heat stress, high irradiance and drought. The specific transcriptomic response to this stress combination in Carrizo was directed to regulate RNA metabolic pathways and translation processes, potentially conferring an advantage with respect to Cleopatra. In addition, we found endoplasmic reticulum stress response as common to all individual and combined stress conditions in both genotypes and identified the accumulation of specific groups of heat shock proteins (HSPs), such as small HSPs and HSP70s, and regulators of the unfolded protein response, BiP2 and PDIL2-2, as possible factors involved in citrus tolerance to triple stress combination. Taken together, our findings provide new insights into the acclimation process of citrus plants to multiple stress combination, necessary for increasing crop tolerance to the changing climatic conditions.

## Introduction

Climate change impact on agrosystems together with other adverse factors directly derived from human activity compromise agricultural production and food security [1, 2]. Thus, the increase in temperatures and extreme weather events (flooding, droughts, heat waves), the aggravation of pest and plant disease incidences, as well as pollution of soil, water and air, cause stressful situations for plants that affect their development, limit their productivity and decline their survival [3–5]. Studying plant responses to adverse situations provides essential information to improve agriculture production through new farming practices and plant breeding ( [6, 7] [8, 9]). In most cases, stress situations do not occur separately, but two or more stressful situations affect the plants at the same time [10]. Although the combination of two stressors can be antagonistic and reduce their impact on the plant, the majority of stress combinations have a synergistic effect, which means that the joint effect of various adverse conditions exceeds that of the sum of the effects of each isolated stress condition [11]. Recent investigations have studied the effects of combined stress on model plants and crops, demonstrating that the combination of two or more stress conditions induces a specific plant response with new traits, different to those induced by the individual stresses [12–15]. However, most of these works include the study of two stress factors, and only a low percentage of these reports shows the interaction of three or more factors [8]. Recent studies have highlighted the importance of studying the effects of multifactorial stress combination (three or more factors acting simultaneously) on plants, since it has been shown that, even when low intensity individual stresses have no significant impact on plant growth, increasing the number of stressors produce a severe impact that causes declines in plant growth and survival [16].

Transcriptomic studies performed to dissect plant responses to stress have shown that two different genetic responses are found: a shared response to individual and combined stress situations, and a unique reaction to stress combination [12–15, 17]. Among shared responses, activation of universal pathways to stress situations include reactive oxygen species (ROS) homeostasis, osmoregulation, DNA repair, unfolded protein response (UPR), hormonal signaling, etc. Examples of specific responses to particular stress combinations are the accumulation of multiprotein bridging factor 1c (MBFc1) and ascorbate peroxidase 1 (APX1) proteins (mediated by abscisic acid) under drought and heat stress conditions [18], and the overexpression of the transcription factors ZINC FINGER 6 and 10 (*ZAT6* and *ZAT10,* mediated by jasmonic acid) under conditions of high irradiance and heat [12]. Moreover, in the cyanobacterium *Synechocystis*, *de novo* synthesis of D1 protein under salt and light stress combined conditions was reduced due to the inhibition at transcriptional and translational levels of *psbA*, its encoding gene, declining, therefore, the repair rate of photosystem II [19].

Carrizo citrange and Cleopatra mandarin are two citrus rootstocks with contrasting tolerance to stress conditions. Carrizo has been described as a vigorous genotype, tolerant to high temperatures, but sensitive to saline, calcareous, and alkaline soils [20]. In turn, Cleopatra is tolerant to drought and salinity but sensitive to high temperatures [20, 21]. In addition, Carrizo and Cleopatra have been studied as citrus model rootstocks for their different physiological and molecular responses to multiple abiotic stress conditions (individual or combined; [21–23]). Here, we performed a transcriptomic and proteomic study in leaves of Carrizo and Cleopatra plants subjected to multifactorial stress combination of drought, high irradiance, and high temperatures, in all possible combinations. Our data show a specific response at both transcriptomic and proteomic levels to each stress combination, which cannot be inferred from individual stresses, in both citrus genotypes.

## Results

### Leaf damage in citrus plants subjected to multiple stress combination

**Figure 1:**
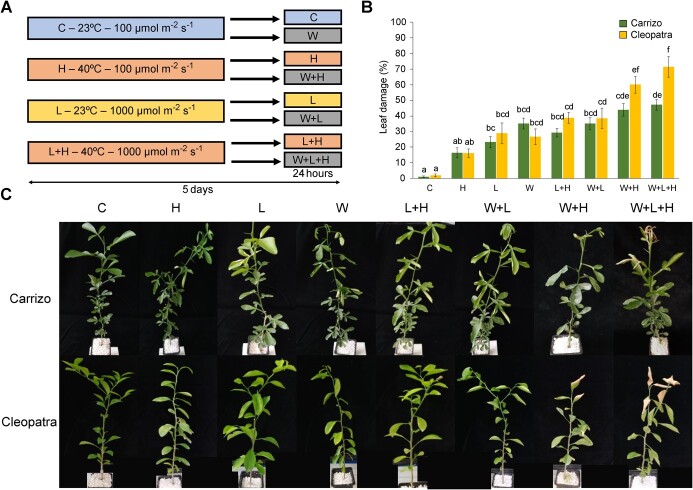
(A) Experiments conducted in Carrizo and Cleopatra plants under control conditions (C), high temperatures (H), high irradiance (L), water stress (W); and combined L + H, W + L, W + H, and W + L + H. (B) Leaf damage in Carrizo and Cleopatra plants under different conditions. (C) Representative images of plants under different conditions. Different letters denote significance at *P* < 0.05. Adapted from Balfagón et al. [23].

Leaf damage in Carrizo and Cleopatra plants subjected to individual and multiple combined abiotic stress conditions was analyzed 24 hours after the end of the different stress treatments (Fig. 1B, C). Heat stress (H) did not trigger significant leaf damage to any citrus genotype compared to control (C) plants. High irradiance (L) and water stress (W), however, induced similar increases in leaf damage with respect to C in both genotypes (ranging between 23% and 35%). L + H and W + L increased the percentage of damaged leaves of Carrizo plants (30% and 35%, respectively) and Cleopatra (39% in both stress conditions). In Cleopatra, W + H had a more pronounced effect on leaf appearance than L + H and W + L, increasing leaf damage to 60%. Finally, under the triple stress combination (W + L + H), Cleopatra reached the highest percentage of leaf damage (71%), whereas Carrizo maintained a percentage of healthy leaves similar to that of the rest of the two-factor stress combinations (Fig. 1B, C).

### Transcriptomic and proteomic responses of citrus plants subjected to multiple stress combination

**Figure 2:**
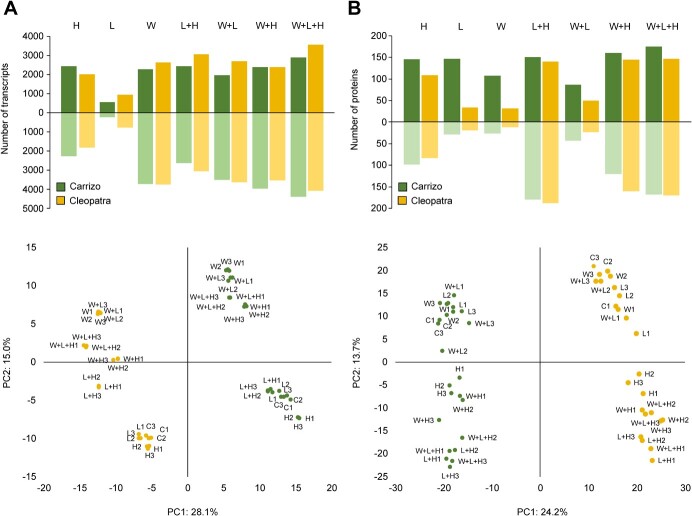
(A) Number of differentially regulated transcripts with respect to control (C; top) and PCA score plot of transcriptomic profile (bottom) in Carrizo and Cleopatra plants subjected to individual and all possible combinations of heat stress (H), high irradiance (L) and water stress (W). (B) Number of differentially accumulated proteins with respect to C (top) and PCA score plot of proteomic profile (bottom) of Carrizo and Cleopatra plants subjected to individual and all possible combinations of H, L, and W.

Transcriptomic and proteomic analyses of Carrizo and Cleopatra plants subjected to the different stress treatments were performed. As shown in Figure 2 and Supplementary Tables S1-S28, the number of differentially regulated transcripts (DRTs) induced by L in both genotypes was lower than that induced by the other stress conditions. On the contrary, W + L + H triggered the highest number of DRTs in both genotypes (Fig. 2A). L, W, and W + L induced a reduced number of differentially accumulated proteins (DAPs), especially in Cleopatra plants, while H, applied individually or in combination with L and/or W, induced marked changes in the number of DAPs in both citrus genotypes (Fig. 2B).

Principal component analyses (PCA) revealed that the main differences among transcriptomic and proteomic data were due to the genotypes (Fig. 2, bottom panels). Thus, the first principal component (PC1), accounting for 28.1% and 24.2% of total variability, divided both genotypes subjected to the different stresses for both transcriptomic and proteomic analyses, respectively. As determined by PC2 for transcriptomic analysis, samples involving W (individually or in combination with L and/or H) clustered together, whereas the rest of stresses (H, L, L + H) were closer to each other. These groupings were more obvious in Carrizo, since Cleopatra L + H samples showed more differences with L, H, or C. Similarly, regulation of transcription factors (TFs) under multiple abiotic stress conditions showed a different expression pattern between Carrizo and Cleopatra in response to the different stresses (Fig. S3 and Supplementary Tables S30–S32). TFs expression profile in Carrizo plants was less altered with respect to C in response to H, L and L + H conditions, whereas in Cleopatra, L + H caused a higher impact on TF accumulation (Fig. S3). All water stress conditions (W, W + L, W + H, and W + L + H) induced a similar TF expression profile in Carrizo, whereas in Cleopatra, the expression profile under W + L + H was different from the rest (Fig. S3A). In addition, the overlap of upregulated and downregulated TFs between both genotypes under W + L + H was around 60% (Fig. S3C). Proteomic PCA results showed a different trend with respect to transcriptomic data. In this sense, L, W, and W + L were closer related to C in both genotypes while samples subjected to high temperatures (H, L + H, W + H and W + L + H) were found to be more distant from the control.

### Common responses to multiple stress combination between Carrizo and Cleopatra plants

**Figure 3:**
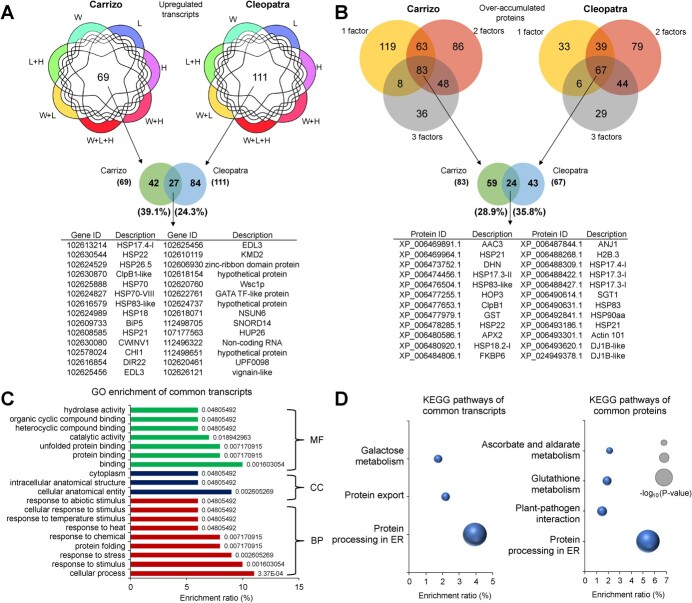
(A) Venn diagrams highlighting the overlap among upregulated transcripts in response to heat stress (H), high irradiance (L), water stress (W), and their combinations in Carrizo and Cleopatra (top). List of the upregulated transcripts common to all stress conditions in both genotypes (bottom). (B) Venn diagrams highlighting the overlap among over-accumulated proteins under 1, 2 or 3 stress factor conditions in Carrizo and Cleopatra (top). List of over-accumulated proteins common to 1, 2, or 3 stress factors conditions in both genotypes (bottom). (C) GO enrichment of common upregulated transcripts to all individual and combined stress conditions in Carrizo and Cleopatra. Numbers in each bar depict P-values. (D) KEGG pathway enrichment of commonly upregulated transcripts to all stress conditions (left) and commonly over-accumulated proteins to 1, 2 or 3 stress factors conditions (right) in Carrizo and Cleopatra. MF, molecular function; CC, cellular component; BP, biological process; ER, endoplasmic reticulum.

Transcriptomic analysis showed that 69 and 111 transcripts in Carrizo and Cleopatra, respectively, were upregulated in response to all individual and combined stress conditions (Fig. 3A). Among these transcripts, only 27 were common between both genotypes (Fig. 3A). According to the gene ontology (GO) enrichment analysis, Molecular Function (MF) enriched terms from these transcripts were “binding,” “protein binding,” or “unfolded protein binding” (Fig. 3C) and the biological process (BP) enriched terms were “response to stimulus,” “response to stress,” and “protein folding.” The Kyoto Encyclopedia of Genes and Genomes (KEGG) pathway “protein processing in reticulum endoplasmic (ER)” was the most significantly enriched among the common upregulated transcripts and proteins between both genotypes (Fig. 3D).

Only five proteins were overaccumulated under all stress factors in Carrizo and none in Cleopatra (Fig. S1). However, 83 proteins in Carrizo and 67 in Cleopatra were over-accumulated under conditions of 1, 2, or 3 stress factors combined (Fig. 3B). Among these common proteins, 24 were common to both genotypes and showed enrichment in the KEGG pathway “protein processing in ER” (Fig. 3D).

Due to the importance of the “protein processing in ER” pathway in response to the different individual and combined abiotic stress conditions, we compared the different regulation of transcripts and proteins related to this pathway in Carrizo and Cleopatra (Fig. S2). In general, stress imposition altered this pathway in both genotypes showing a similar number of DRTs involved in this pathway. Individual and, specially, combined stress conditions involving L (L, L + H, W + L, and W + L + H) triggered the upregulation of transcripts related to this pathway in both genotypes (Fig. S2A). In turn, heat stress conditions (H, L + H, W + H, and W + L + H) induced a higher number of over-accumulated proteins than that in response to W, L, and W + L. Furthermore, Carrizo showed a higher number of DAPs compared to Cleopatra under L, W, L + H, W + L, and W + L + H. Among the upregulated transcripts related to the “protein processing in ER” pathway in response to three stress factors combination, 38 were shared between Carrizo and Cleopatra, and 9 and 7, respectively, were specific to each genotype. In turn, 12 of 25 over-accumulated proteins in Carrizo under W + L + H were not found among the over-accumulated proteins in Cleopatra in response to triple stress, and only four were over-accumulated in Cleopatra and not in Carrizo.

### Unique transcriptomic and proteomic response to triple stress combination

**Figure 4:**
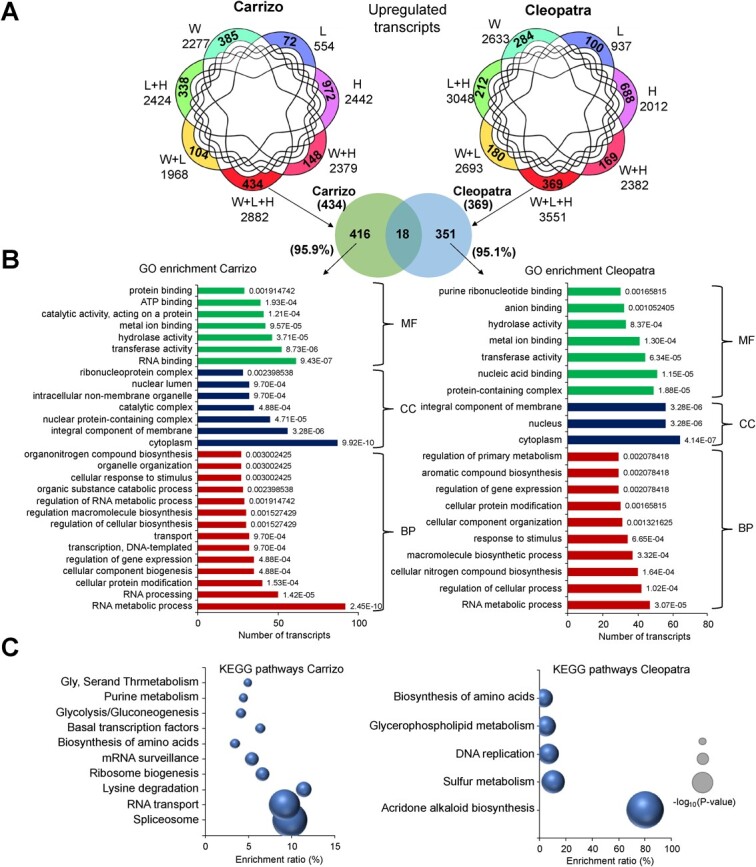
(A) Venn diagrams highlighting the overlap among upregulated transcripts in response to heat stress (H), high irradiance (L), water stress (W) and their combinations (top). Venn diagram highlighting the overlap among transcripts specifically upregulated in Carrizo and Cleopatra plants subjected to W + L + H (bottom). (B) GO and (C) KEGG pathway enrichment of specific upregulated transcripts in Carrizo or Cleopatra plants subjected to W + L + H. Numbers in each bar in (B) depict *P* values. MF, molecular function; CC, cellular component; BP, biological process.

**Figure 5:**
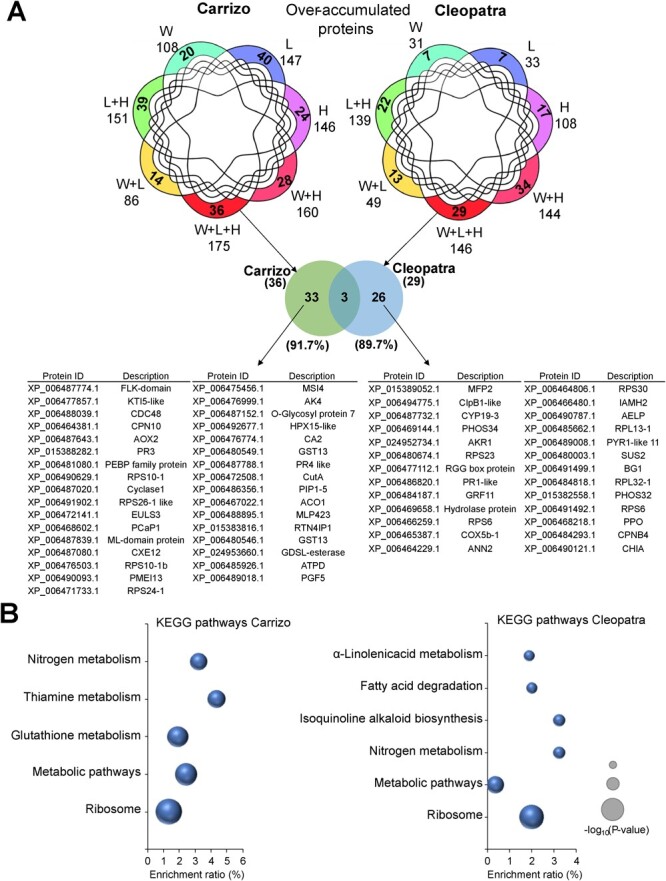
(A) Venn diagrams highlighting the specific over-accumulated proteins in response to heat stress (H), high irradiance (L), water stress (W), and their combinations (top). Venn diagram highlighting overlap among proteins specifically over-accumulated in Carrizo and Cleopatra plants subjected to W + L + H (middle). List of over-accumulated proteins specific to Carrizo or Cleopatra plants under W + L + H (bottom). (B) KEGG pathway enrichment of specific over-accumulated proteins in Carrizo (left) and Cleopatra (right) plants subjected to W + L + H.

The specific transcriptomic and proteomic response to W + L + H was analyzed in both citrus genotypes. As shown in Figures 4 and 5, 434 and 369 transcripts were specifically upregulated under W + L + H conditions in Carrizo and Cleopatra, respectively. However, only 18 of these transcripts overlapped between Carrizo and Cleopatra, and more than 95% of them were specific to each genotype (Fig. 4A and Supplementary Table S29). GO term analyses revealed that, among transcripts specifically upregulated by W + L + H, the BP “RNA metabolic process” was the most enriched term in both genotypes, although the number of transcripts in Carrizo almost doubled that of Cleopatra (92 and 47, respectively). Carrizo also showed other enriched BP terms related to RNA, such as “RNA processing” or “regulation of RNA metabolic process”, as well as the MF term “RNA binding” (Fig. 4B). KEGG pathway analysis of the specific upregulated transcripts revealed that the most enriched terms in Carrizo were “Spliceosome” and “RNA transport” pathways, and the “acridone alkaloid biosynthesis” pathway in Cleopatra (Fig. 4C).

Among the unique over-accumulated proteins under W + L + H, 33 were specific to Carrizo, 26 to Cleopatra, and only three were common between both genotypes (Fig. 5A). In both genotypes, the most enriched KEGG pathway terms among these proteins were “ribosome” and “metabolic pathways” (Fig. 5B).

### Analysis of the translation pathway in citrus plants subjected to multiple stress combination

**Figure 6:**
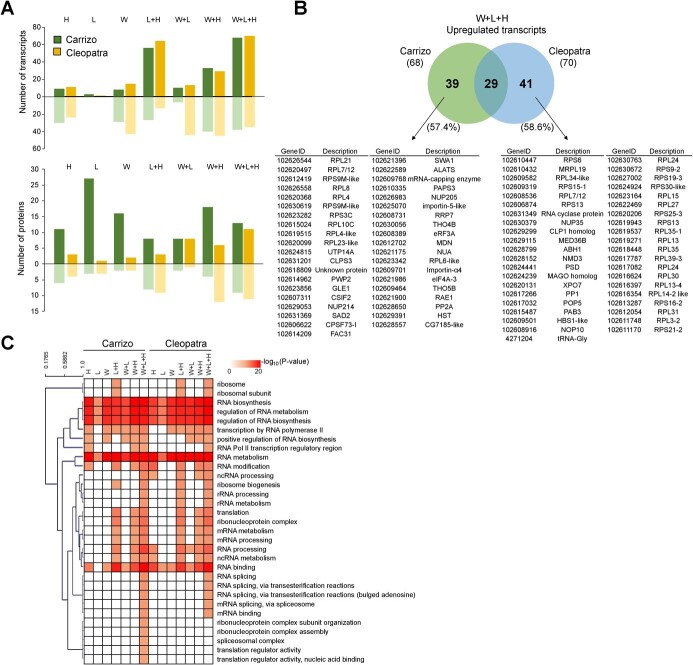
(A) Number of differentially regulated transcripts (top) and accumulated proteins (bottom) that belong to the translation pathway in response to heat stress (H), high irradiance (L), water stress (W), and their combinations. (B) Venn diagram highlighting the overlap among upregulated transcripts related to the translation pathway in Carrizo and Cleopatra plants subjected to W + L + H. (C) Clustered heat map of GO enriched terms related to translation process, RNA metabolism, and ribosome regulation in Carrizo and Cleopatra plants subjected to individual and combined stress conditions.

Transcriptomic and proteomic regulation related to the translation pathway showed differences among stress treatments and genotypes (Fig. 6). Individual stresses (particularly L) caused a reduced alteration in the number of DRTs than that of combined stresses in both genotypes, except for W + L. L + H, W + H, and W + L + H induced the highest number of DRTs in Carrizo and Cleopatra. When considering upregulated transcripts, L + H and W + L + H were the most impacting conditions (Fig. 6A). At the proteomic level, the number of Carrizo DAPs was higher than that in Cleopatra under all stress conditions except for W + L + H (with similar number of DAPs in both genotypes). In addition, under all stress conditions, with the exception of W + L, the number of over-accumulated proteins was higher in Carrizo than in Cleopatra (Fig. 6A). Of the total upregulated transcripts under W + L + H, 57.4% and 58.6% of them were specific to Carrizo and Cleopatra, respectively (Fig. 6B). GO enrichment analysis of translation process, RNA metabolism and ribosome transcripts is shown in Figure 6C. A cluster of GO terms was enriched for all stress conditions in both genotypes (“RNA biosynthetic process,” “regulation of RNA metabolic process,” “regulation of RNA biosynthetic process,” “RNA metabolic process”), and other clusters indicated the enrichment of GO terms in response to W + L + H and L + H and/or W + H conditions (“ribosome biogenesis,” “rRNA processing,” “rRNA metabolic process,” “translation,” “ribonucleoprotein complex,” “mRNA metabolic process,” “mRNA processing”). Finally, five GO terms related to RNA splicing and mRNA binding were specific to the W + L + H stress conditions in both genotypes, and other five GO terms related to the ribonucleoprotein complex, spliceosomal complex, and translation regulator activity were only enriched in Carrizo plants under the triple stress combination.

## Discussion

Previous research analyzing the response of citrus plants to multiple abiotic stress conditions showed that water stress, high temperatures, and high irradiance, applied in all possible combinations, caused a severe impact on plant survival and photosynthetic processes [23]. However, Carrizo was more tolerant than Cleopatra when high temperatures occurred together with water stress or high irradiance. This may be due to the better stomatal and transpiration regulation of Carrizo leaves that contribute to reducing heat stress pressure [21, 23]. In addition, other factors that contribute to the enhanced tolerance of Carrizo include a higher activation of the antioxidant system under combinations of drought and heat stress [24], and better photosynthetic apparatus repair during combined conditions of high irradiance, heat stress, and drought [23]. However, a transcriptomic and proteomic study analyzing the different stress pathways activated under multifactorial stress combination in two citrus genotypes with contrasting ability to tolerate combined stress conditions was not reported yet. In this study, a transcriptomic and proteomic analysis in Carrizo and Cleopatra plants subjected to multifactorial stress combination of high irradiance, heat stress, and drought was conducted.

Transcriptomic analysis showed a similar pattern of response to the individual and combined stresses between Carrizo and Cleopatra. In both genotypes, water stress was the most determinant condition to modulate transcriptome with respect to control conditions (Fig. 2A). In particular, W + L + H was the condition that induced a higher number of DRTs (including TFs regulation), although significant differences were appreciated between Carrizo and Cleopatra in the expression profile PCA and the TFs regulation (Fig. 2A and 3SC). Individual L and H were the conditions that less affected gene expression patterns in both genotypes (Fig. 2 and S3). However, L + H induced more transcriptomic changes in Cleopatra than in Carrizo, which indicates a genotype-specific response to this stress combination. Cleopatra is more sensitive to water or light stress in combination with high temperatures due to a lower antioxidant capacity and the higher damage to the photosynthetic apparatus [23, 24]. These results indicate that, whereas the effects of L and H conditions applied individually were negligible for both genotypes, Cleopatra was more sensitive to the L + H stress combination and induced a stronger transcriptomic response compared to Carrizo (Fig. 2A and S3). Despite the impact of water stress on the transcriptome, heat stress (L + H, W + H, and W + L + H) was the condition that caused more changes in the proteome of citrus plants. Several studies have reported specific transcriptomic responses to two combined stress factors that were not observed under stresses applied individually in Arabidopsis [12, 14, 15] and other plant species [17, 25, 26]. However, very few studies showed the transcriptomic changes in response to three or more stress factors in combination [8]. Prasch and Sonnewald [13] showed that the specific transcriptomic response of Arabidopsis plants to the combination of three stress factors (high temperatures, water stress, and virus infection), different than that in response to one or two stress factors, was a determinant for plant tolerance to the triple stress. More recently, a transcriptomic analysis performed in Arabidopsis plants under multiple abiotic stress combinations applied in a factorial manner (six in total) showed that each multifactorial stress condition caused a specific transcriptomic fingerprint [16]. Here, we identified a set of transcripts specifically upregulated in response to W + L + H in both Carrizo and Cleopatra. However, transcriptomic and proteomic differences between both genotypes in response to W + L + H could explain the higher tolerance of Carrizo compared to Cleopatra. The lack of correlation between transcriptomic and proteomic responses suggests an important role for translational regulation in the response to stress. Carrizo showed a stronger upregulation of RNA processing GO terms (“RNA metabolic process,” “RNA processing,” “RNA binding,” “regulation of RNA metabolic process,” or “ribonucleoprotein complex”) and KEGG pathways related to RNA metabolism, translation, and protein synthesis (“spliceosome,” “RNA transport,” “ribosome biogenesis,” mRNA surveillance,” or “biosynthesis of amino acids”) (Fig. 4). These data indicate that Carrizo showed a differential response to W + L + H with respect to Cleopatra directed to regulate RNA metabolism, translation processes and protein formation. RNA metabolism, post-transcriptional and translation regulation are important mechanisms to control abiotic stress-related responses at gene expression and proteomic levels [27–29]. Although the number of DRTs related to translation pathways was similar between Carrizo and Cleopatra under W + L + H, more than half of the induced transcripts were different between genotypes (Fig. 6B). Triple stress combination induced a higher number of DRTs than any other stress combination (Fig. 6A), and the analysis of the GO-enriched terms from translation and RNA metabolic processes showed a higher upregulation of transcripts involved in RNA processing in both genotypes (Fig. 6C). However, Cleopatra plants triggered more transcripts related to ribosome and ribosomal subunits whereas, in Carrizo, more DRTs were involved in translation regulation and assembly of spliceosomal and ribonucleoprotein complexes under W + L + H conditions (Fig. 6B). One of these genes was the eukaryotic initiation factor 4A-3 (eIF4A-3) which belongs to a group of helicases that are key in the process of initiation of translation [30]. Regulation and functionality of eIF4A may be important in plant tolerance to abiotic stresses [29]. Two reports showed that the overexpression of the pea gene eIF4A in rice or tobacco resulted in a higher tolerance to salt stress [31, 32]. Also, overexpressing a Na+/H+ antiporter (NHX1) and eIF4A in Arabidopsis induced better plant development and ROS scavenging compared to wild type [33]. Our results suggest that the activation of regulators of mRNA metabolism and translation, including eIF4A-3, may be a key component to acclimate to harsh stress situations originated by the combination of three adverse conditions and could confer an advantage to Carrizo.

The identification of common signaling pathways and responses to multiple abiotic stress conditions is an interesting strategy for developing improved crop varieties [8]. The transcriptomic and proteomic analyses performed in this study show, for the first time, common pathways activated in response to high temperatures, high irradiance, drought, and all their possible combinations in two citrus genotypes. The differences in these pathways between Carrizo, more tolerant to the multiple stress combination (Fig. 1; [23]), and Cleopatra, provide valuable information about potential strategies of citrus plants to cope with these stress conditions that can be used in future breeding programs. In both genotypes, the application of any individual or combined stress condition induced the upregulation of transcripts related to protein folding and protein processing in ER, such as HSPs and other chaperones, or UPR regulators (Fig. 3). Similarly, most of the over-accumulated proteins common to one, two, or three stress factor conditions in both genotypes were HSPs and components of the UPR (Fig. 3). By analyzing the transcriptomic and proteomic regulation of “protein processing in ER” pathway under W + L + H, we observed that more than 80% of the upregulated transcripts were common between Carrizo and Cleopatra (Fig. S2A), but 48% of the overaccumulated proteins of this pathway in Carrizo under W + L + H were not found in Cleopatra (Fig. S2B). These results suggest that the differential proteomic response to the triple stress combination between both genotypes could explain, at least in part, the higher tolerance of Carrizo to stress combination. Among the differential proteins accumulated in response to the triple stress, a disulfide isomerase-like protein (PDIL2-2) and a chaperone-binding protein (BiP2) were found (Fig. S2B). These proteins are part of the UPR and their overaccumulation are linked to enhanced resistance to abiotic stress conditions [34–36]. In addition, different types of heat shock proteins (HSPs), mainly from the families of small HSP (sHSP) and HSP70s were differentially accumulated in Carrizo under the different stresses. The importance of these proteins in ER stress and, in general, in plant tolerance to multiple stress conditions has been widely studied ( [37]; [38]). sHSPs capture unfolded proteins to prevent irreversible aggregation and facilitates protein disaggregation together with other HSPs. Their accumulation under heat stress is associated with the protection of photosystem II in chloroplasts [39]. Therefore, this stress-specific role of HSPs could partially explain the better performance of Carrizo photosynthetic apparatus under multiple abiotic stress combinations with respect to Cleopatra [23]. HSP70s are important for mitigating the effects of multiple stress conditions. These proteins prevent aggregation and assist refolding of non-native proteins, translocate unfolded or misfolded proteins out of the ER to facilitate their degradation, and are involved in the intracellular movement of proteins [40]. In addition, sHSP and HSP70 have been reported to have a role in maintaining translation processes during heat stress [41, 42]. Therefore, our results suggest that maintaining active UPR regulators and HSPs could be crucial for citrus tolerance to multifactorial stress combination, acting as good candidates for future programs of plant breeding.

In agriculture, rootstocks are used to improve variety productivity by conferring agronomic qualities, such as, among others, tolerance to stress conditions [20, 43, 44]. This study shows, for the first time, the differences at transcriptomic and proteomic levels of two genotypes with contrasting tolerance to multiple abiotic stress factors. Here, it has been demonstrated that protein processing in ER is a common pathway to all individual and combined situations of water stress, high temperatures and high irradiance, and the accumulation of sHSP, HSP70, PDIL2-2, and BiP2 proteins are probably linked to an enhanced tolerance of citrus plants to stress combination. In addition, upregulation of the RNA metabolism and translational processes could be a key response of Carrizo plants to better tolerate the combination of water stress, high temperatures, and high irradiance. This study reveals new and valuable information for the improvement of citrus industry, aimed at minimizing the negative effect of multiple stressors on citrus plants that are already facing the effects of global climate changes.

## Materials and methods

### Plant material and growth conditions

Twelve-month-old seedlings of Carrizo citrange (*Poncirus trifoliata* L. Raf. x *Citrus sinensis* L. Osb.) and Cleopatra mandarin (*Citrus reshni* Hort. Ex Tan.) were acquired from Beniplant, a plant nursery (Peñíscola, Spain), transferred to plastic pots filled with perlite and placed in a greenhouse with natural photoperiod and 25.0 ± 3.0°C during the day and 18.0 ± 3.0°C during the night). Watering of seedlings was performed every other day with 0.5 L nutritive solution adapted to citrus [23]. To allow plant acclimation, 2 weeks before starting the stress treatments, plants were transferred to climatic chambers at 23°C, with 16 hours of light (100 μmol∙m^−2^∙s^−1^ intensity).

### Stress treatments

Acclimated plants of were subjected to individual conditions of heat stress (H), high irradiance (L) and water stress (W); and to combined conditions of high irradiance and heat stress (L + H), water stress and high irradiance (W + L), water stress and heat stress (W + H) and water stress, high irradiance, and heat stress (W + L + H). H was applied by raising temperature to 40°C during 5 days. L was imposed by increasing light intensity to 1000 μmol∙m^−2^∙s^−1^ from 12 to 20 hours (8 hours a day) for 5 days. W was imposed by changing pot substrate by dry perlite 24 hours on Day 5 (Fig. 1A). This stress treatment allows the application of the three adverse conditions simultaneously in a systematic way, obtaining a representative response of the plant to each of them. Percentage of healthy leaves was measured similar to Balfagón et al. [12, 23]. Green leaves were taken as healthy leaves, and chlorotic or wilted leaves were considered as damaged leaves. All stress treatments were performed in parallel with at least 10 plants per stress treatment. Fully expanded leaves with an intermediate position in the canopy were harvested and immediately frozen with N_2_ for use in subsequent analysis.

### Proteomic analysis

300 mg of leaves (fresh weight) were used for comparative proteomic analyses in three biological replicates. Total protein extraction and mass spectrometry analysis were performed as described in [45]. To be considered as a differentially accumulated protein (DAP), log_2_ value of their fold change (FC) should be over 0.6 or under −0.6, according to Student's *t* test (two-tailed; *P* < 0.05) and results repeated in the three replicates. OmicsBox (https://www.biobam.com/omicsbox) and UniProtKB (https://www.uniprot.org) were used for functional annotation. The protein sequences were submitted to a BLAST search against the NCBI nonredundant green plant protein database (taxa: 33090, Viridiplantae).

### RNA-Seq analysis

RNA-seq analysis was performed as described in Balfagón et al. [23]. Functional annotation analyses were performed using the same programs than in the proteomic analysis. The Multiple Experiment Viewer (MeV) software v. 4.9.0 [46] was used for creating heat maps.

### Statistical analysis

All experiments were repeated at least three times. Two-way ANOVA followed using a Tukey *post hoc* test (*P* < 0.05) was used for leaf damage measurements. Functional annotation and enrichment analysis (Fisher's exact test *P* < 0.05) of GO terms were conducted using Omicsbox software. KEGG pathway enrichment analysis was performed using KOBAS-i [47]. Venn diagram overlaps were subjected to hypergeometric testing using the package phyper (https://stat.ethz.ch/R-manual/R-devel/library/stats/html/Hypergeometric.html) in R software environment. PCA was performed by means of the Soft Independent Modeling of Class Analogy software v. 13.0.3.0, using the log_2_ transformed data and unit variance normalization.

## Acknowledgments

This work was supported by Grants PID2019-104062RB-I00 and TED2021-129795B-I00 funded by MCIN/AEI/10.13039/501100011033 and by the European Union- NextGenerationEU. Funding was also obtained from Universitat Jaume I (UJI-B2022-18) and Generalitat Valenciana (CIAICO/2021/063). DB was supported by the European Union–NextGenerationEU and the Ministerio de Universidades (MGS/2021/17). S.I.Z. was supported by MCIN (RYC2020-029967-I). T.R.O. was supported by the Fundação Carlos Chagas Filho FAPERJ (E-26/204.192/2021) and Coordenação de Aperfeiçoamento de Pessoal de Nível Superior (CAPES–001).

## Author Contributions

D.B., S.I.Z., and A.G.C. conceived the research plan and designed the experiments. D.B. and T.R.O. performed the experiment, harvest plan material, and analyzed samples. T.R.O. and C.S.C. performed proteomic analysis. A.G.C. supervised the project and provide funding. D.B., S.I.Z., and A.G.C. wrote the manuscript and prepare figures. All authors read and approved the final version of the manuscript.

## Data availability

RNA-Seq data files were deposited in GEO (https://www.ncbi.nlm.nih.gov/geo/) under the following accession number GSE203331. Mass spectrometry proteomic data were deposited with ProteomeXchange consortium via the PRIDE (https://www.ebi.ac.uk/pride/) partner repository with the data set identifier PXD034419.

## Conflict of interest statement

None declared.

## Supplementary Data

Supplementary data is available at Horticulture Research online.

## Supplementary Material

Web_Material_uhad107
