# Supplementary figures and images for: Omics analyses in citrus reveal a possible role of RNA translation pathways and Unfolded Protein Response regulators in the tolerance to combined drought, high irradiance, and heat stress
Source: Hortic Res. 2023 May 19;10(7):uhad107. doi: 10.1093/hr/uhad107 (PMC10419850; doi:10.1093/hr/uhad107)

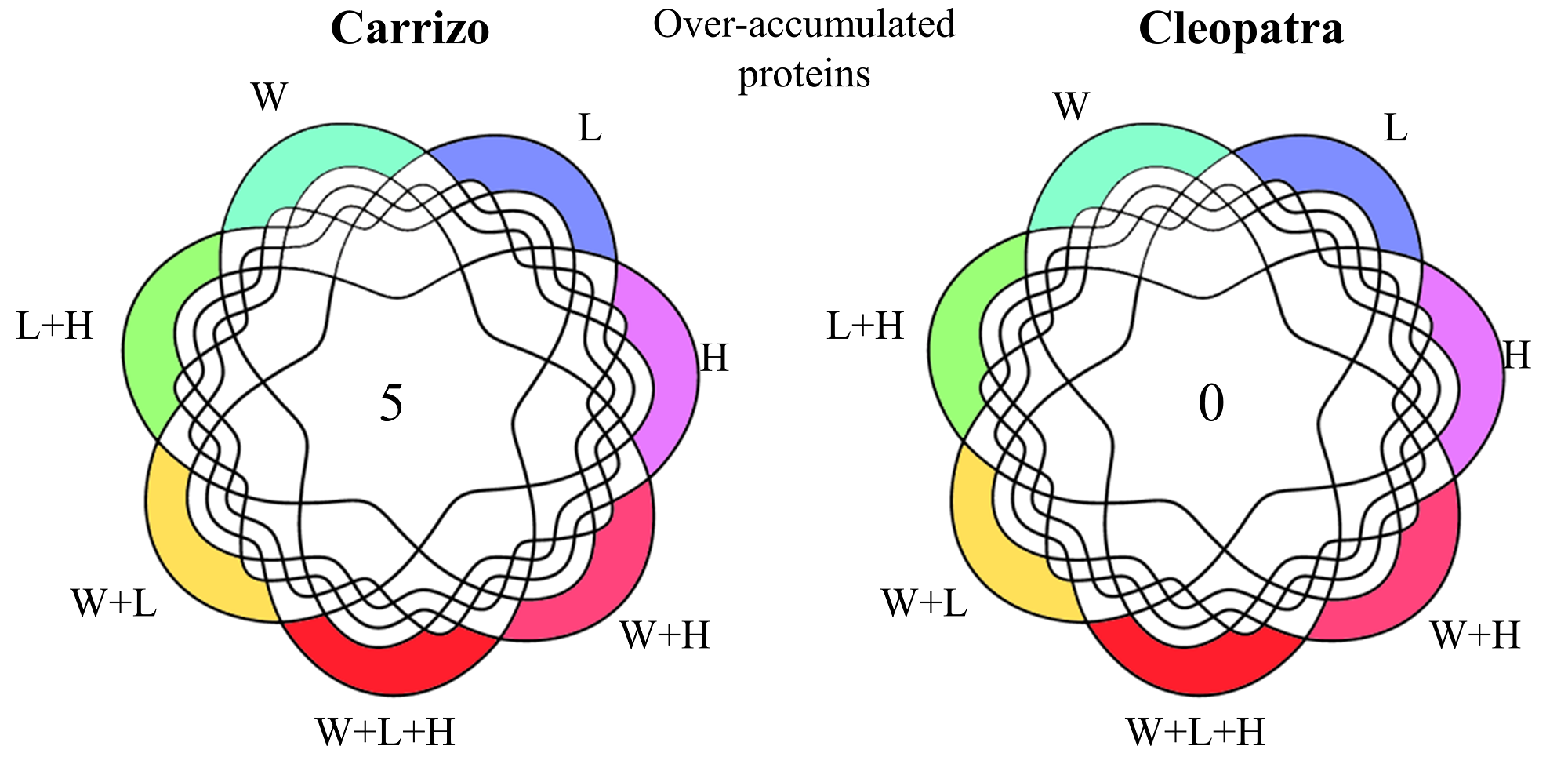

Supplement: Web_Material_uhad107 [file web_material_uhad107.zip › Figure S1.tif]

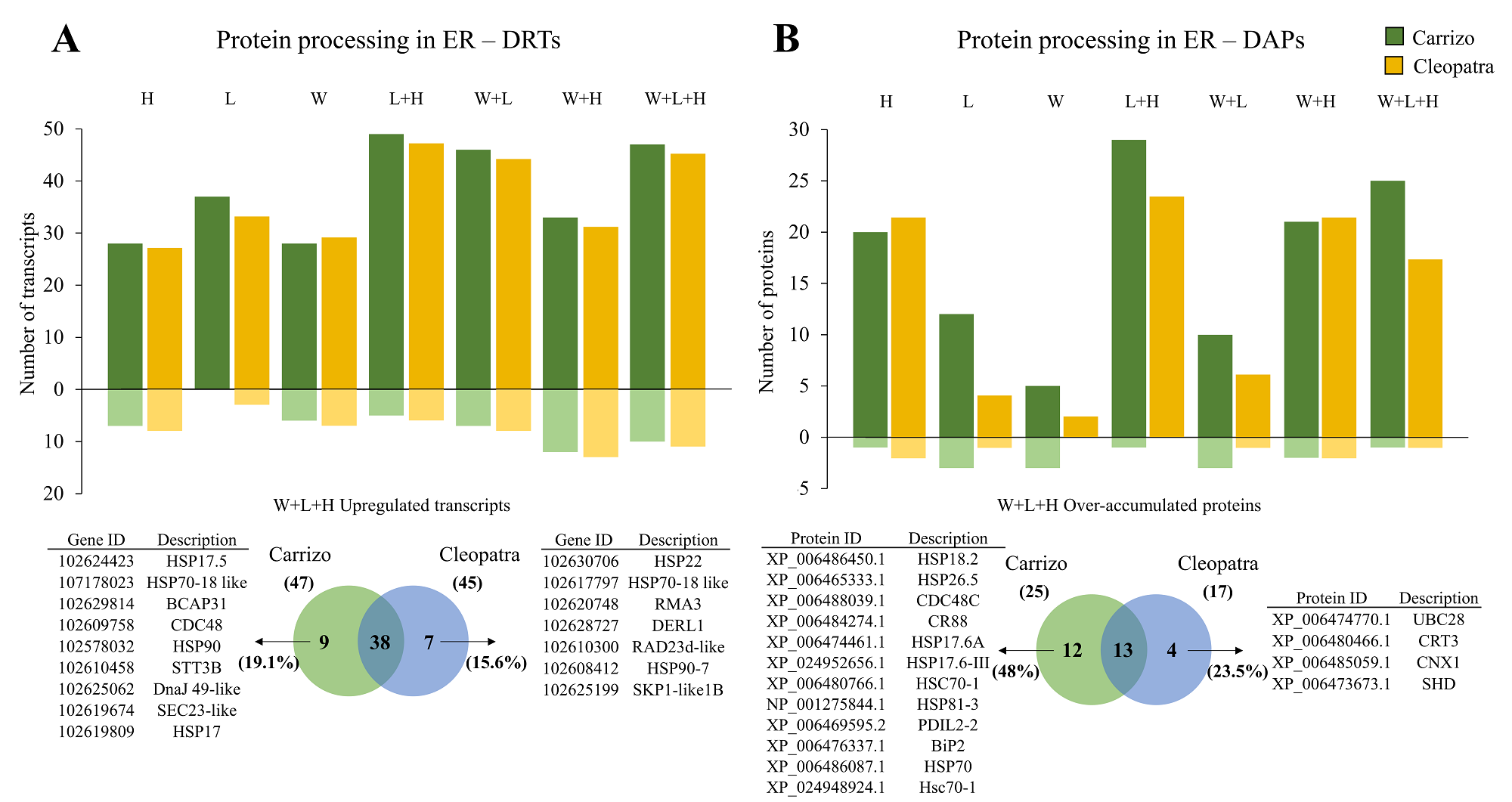

Supplement: Web_Material_uhad107 [file web_material_uhad107.zip › Figure S2.tif]

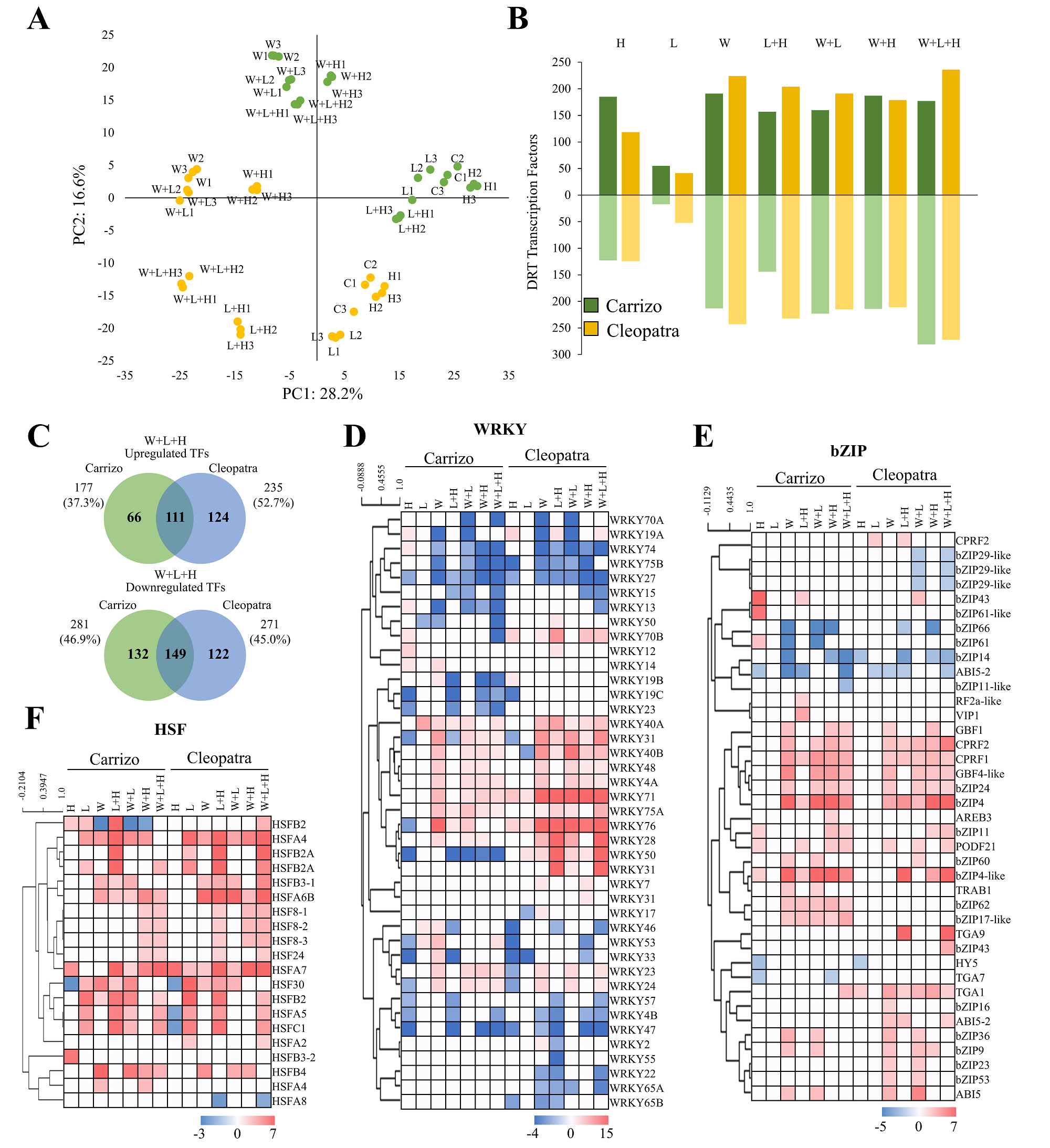

Supplement: Web_Material_uhad107 [file web_material_uhad107.zip › Figure S3.tif]
